# The necessity to choose causes reward-related anticipatory biasing: Parieto-occipital alpha-band oscillations reveal suppression of low-value targets

**DOI:** 10.1038/s41598-017-14742-w

**Published:** 2017-10-30

**Authors:** Anna Heuer, Christian Wolf, Alexander C. Schütz, Anna Schubö

**Affiliations:** Experimental and Biological Psychology, Philipps-University Marburg, Marburg, Germany

## Abstract

Positive outcome of actions can be maximized by choosing the option with the highest reward. For saccades, it has recently been suggested that the necessity to choose is, in fact, an important factor mediating reward effects: latencies to single low-reward targets increased with an increasing proportion of interleaved choice-trials, in which participants were free to choose between two targets to obtain either a high or low reward. Here, we replicate this finding for manual responses, demonstrating that this effect of choice is a more general, effector-independent phenomenon. Oscillatory activity in the alpha and beta band in the preparatory period preceding target onset was analysed for a parieto-occipital and a centrolateral region of interest to identify an anticipatory neural biasing mechanism related to visuospatial attention or motor preparation. When the proportion of interleaved choices was high, an increase in lateralized posterior alpha power indicated that the hemifield associated with a low reward was suppressed in preparation for reward-maximizing target selection. The larger the individual increase in lateralized alpha power, the slower the reaction times to low-reward targets. At a broader level, these findings support the notion that reward only affects responses when behaviour can be optimized to maximize positive outcome.

## Introduction

Human behaviour is shaped by the motivation to optimize interaction with the environment to maximize the positive outcome of decisions and corresponding actions. A decision frequently required is where to look next and, as a precursor thereto or consequence thereof, which visual information to select for further processing. As processing resources are limited, the decision which parts of the vast amount of available visual information are selected to guide further behaviour is critical. A way to maximize positive outcome in attentional selection, is to choose the option with the highest value. The value of different options can be learned in an associative manner: When a stimulus co-occurs with a rewarding outcome (e.g., monetary or social reward), a stimulus-reward association is established and the stimulus receives prioritized processing^1^. Indeed, value has been shown to profoundly influence visual selection in human and non-human primates: Parameters of saccadic eye movements such as latency or peak velocity depend on whether or not these movements are associated with the expectation of reward^2–7^. Reward also exerts a powerful influence on covert visual selection (i.e., selective attention) in the absence of any overt eye movements^1,8–11^.

A common feature of studies demonstrating a strong impact of reward on visual selection is that in these tasks, a choice was required to be made between different options varying in reward value. Thus, value was behaviourally relevant because the outcome of behaviour could be optimized by choosing the option with the highest associated reward. A recent study indicates that this necessity to choose is an important factor mediating effects of reward. Wolf, Heuer, Schubö and Schütz (under review) examined saccade latencies to single targets presented in the left or right hemifield (single-trials). One hemifield was associated with a high reward and the other with a low reward, which participants received for correct responses. A second trial type, in which participants were free to choose between two targets presented to the left and right from fixation to receive the corresponding reward (choice-trials), was interleaved with single-trials. The proportion of these choice-trials in a block of trials was systematically varied, and performance in single-trials was analysed as a function of choice-trial proportion in the same block. Saccade latencies in single-trials were modulated by reward only when choice-trials were present: Saccades to low-reward single targets were delayed, and the magnitude of this delay increased with the proportion of interleaved choice-trials. This effect was not affected by the frequency of saccades into either direction and depended on spatial reward associations in choice-trials rather than in single-trials. These results indicate that reward value affects saccade preparation only when it is behaviourally relevant, for example because a choice needs to (and can) be made that allows maximizing positive outcome by selecting the target with the highest value.

It is not clear, at which level the necessity to choose biases processing in a reward-related manner. The effects could for instance be brought about by a bias in the deployment of spatial attention, in motor preparation or in the sensory processing of the target. Depending on the processing level, the modulations by reward could differ between effectors, as shown previously for eye movements and reaching^12,13^.

In the current study, we set out to test whether the mediation of reward effects by the necessity to choose generalizes to similar actions with other effectors (manual responses), and to identify the processing level at which a reward-related bias is implemented via the neural mechanisms underlying these effects. To this end, we modified the paradigm from Wolf *et al*. (under review): Instead of executing a saccade to the target, participants responded with left or right manual keypresses to the left or right targets, respectively, and the EEG was recorded while they performed the task.

Reward has been shown to affect different stages of processing, for instance early visual processing and the deployment of visual attention^10,14,15^, or post-perceptual stages such as goal-directed action^16,17^. In the present study, we focused on how reward affects processing already in the pre-stimulus interval. We hypothesized that this would be the time at which the modulation of reward effects by the necessity to choose would take effect. For one, the proportion of choices to be made is a more global context factor, which is not varied on a trial-by-trial basis, but constant for larger sets of trials. Thus, we reasoned that a higher choice-trial proportion would result in anticipatory biasing mechanisms engaged in the expectation of reward-associated stimuli, preparing for reward-maximizing processing and response to the target(s). Moreover, an anticipatory bias would be in line with the findings of Wolf *et al*. (under review): Modelling saccade latency distributions indicated that the delay in responses to low-reward targets with higher proportions of choice-trials was due to a reduced baseline level in the response signal. An adjustment of baseline activity would be expected to occur prior to stimulus onset. Therefore, a preparatory period was established in the present paradigm: a change of fixation cross size signalled target onset in a fixed amount of time. We considered two potential types of bias that might take effect in this preparatory period and be reflected in pre-stimulus oscillatory brain activity.

First, there might be an attentional bias in the spatial domain, resulting in suppression of the visual hemifield associated with a low reward. To examine this possibility, we analysed posterior oscillatory activity in the alpha-band (8–14 Hz), which has been identified as a robust correlate of the preparatory allocation of selective attention^18^. Several studies examined oscillatory activity in the interval between a cue and a target (presented with or without distractors), and revealed changes in posterior alpha power consistent with an involvement in anticipatory selective attention. When stimuli are presented in a lateralized fashion, a decrease in alpha power can be observed in the hemisphere contralateral to the target^19–24^. This alpha decrease over parieto-occipital cortex is thought to reflect a facilitation or release from inhibition leading to increased excitability of cortical areas processing the expected visual information^18^. Alpha power does not only reflect enhancement of relevant stimuli, but also suppression of irrelevant stimuli. When targets are presented along with task-irrelevant, distracting information, an increase in alpha can be observed contralateral to the stimuli that are to be ignored or selected against^21,25–28^. The relative alpha increase does not simply arise from alpha desynchronization of the other hemisphere processing the to-be-attended stimuli, reflecting an ‘idle’, passive state, but it reflects an active suppression mechanism^26,29^. This notion is also consistent with the scalp distribution of inhibitory alpha activity: its focus has been shown to track the anticipatory orienting of attention in a spatially specific manner, moving retinotopically with the location of potentially distracting information^27^.

Second, a bias might be implemented at the level of motor preparation. In our task, each hemifield that was assigned a specific reward (low versus high) was coupled to a specific effector (left or right index finger) for large sets of trials. Thus, it is conceivable that lateralized motor preparation occurred with higher proportions of choice-trials. Motor preparation has been shown to reflect in oscillatory activity in the beta-band (15–30 Hz): Prior to movement execution, beta power decreases over sensorimotor cortex at central electrode sites, with a stronger decrease contralateral to the effector^30–34^. Specifically, it has been suggested that the preparatory lateralized change in beta power relates to early motor selection, seeing as it is only observed when informative cues allow for anticipation of the effector to be moved^31^, and can predict participants’ choice well before a button press^30^. When a task required only one single effector, the decrease in beta power during motor preparation scaled with the degree of uncertainty about the upcoming direction of a movement^35^. It has further been shown that these changes in beta power correlate with changes in reaction times^31,35^.

We used an adapted version of the paradigm by Wolf *et al*. (under review) to test whether choice-mediated effects of reward generalize to manual responses, and examined oscillatory brain activity in the alpha and beta band to identify potential underlying biasing mechanisms in the preparatory period preceding target presentation. At the behavioural level, we expected the same pattern of results as in Wolf *et al*. (under review), namely increasing response times to low-reward targets with increasing choice-trial proportion (0 vs. 0.25 vs. 0.75). A lateralization of posterior alpha power with respect to the low-reward hemifield would be taken to suggest a bias in anticipatory selective attention, whereas lateralized beta power at centrolateral electrode sites would indicate a bias in motor preparation. Additionally, early event-related potentials following target presentation were examined to check whether the modulation of reward effects by the necessity to choose would also reflect in lateralization of post-stimulus sensory processing.

## Results

### Behavioural measures

**Figure 1:**
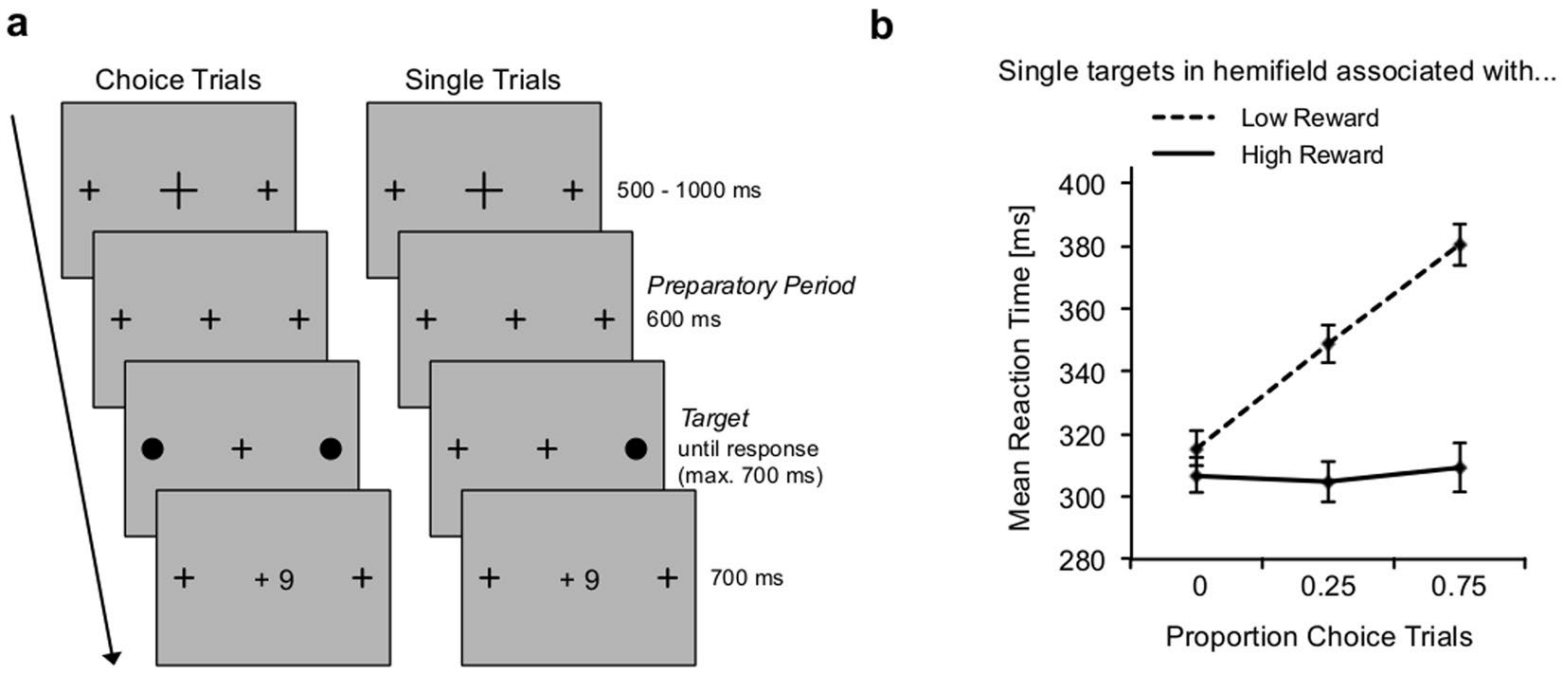
Task and behavioural results. (**a**) Trial procedure for choice-trials (left) and single-trials (right). A trial started with the presentation of a fixation cross (500–1000 ms), which then changed its size to that of the placeholders presented to the left and right, indicating the onset of the target(s) in 600 ms (*preparatory period*). In single-trials, one target replaced one of the placeholders, and participants were to indicate whether the target was presented to the left or right. In choice-trials, both placeholders were replaced by targets, and participants were free to choose either one. The target was present until response, or for a maximum of 700 ms. In each block of trials, one hemifield was associated with a high (9 points) and the other with a low reward (1 point). In correct single-trials, participants received the corresponding reward. In choice-trials, participants received the reward associated with the chosen target. (**b**) Mean reaction times in single-trials as a function of the proportion of choice-trials in the same block. The dashed line represents reaction times to targets presented in the hemifield associated with low reward, the solid line reaction times to targets in the highly-rewarded hemifield. Error bars show standard errors of the means.

The primary measure of interest was reaction time in single-trials, shown in Fig. 1b separately for the three choice-trial proportions and for low- and high-reward targets. Reaction times were slower for low-reward targets than for high-reward targets (F_(1,25)_ = 297.71, p < 0.001, partial ƞ^2^ = 0.923), and for higher choice-trial proportions (F_(2,50)_ = 74.47, p < 0.001 partial ƞ^2^ = 0.75). Importantly, an interaction between choice-trial proportion and reward magnitude (F_(2,50)_ = 130.36, p < 0.001, partial ƞ^2^ = 0.84, ɛ = 0.723) revealed that the reaction time difference between high- and low-reward targets increased with increasing choice-trial proportions. This was due to a slowing of responses to low-reward targets with higher proportions of choice-trials (F_(2,50)_ = 211.98, p < 0.001, partial ƞ^2^ = 0.90). There was no effect of choice-trial proportion on reaction times to high-reward targets.

Accuracy in single-trials was near perfect (97.04% ± 0.47%) and mirrored the pattern of reaction times, confirming that there was no speed-accuracy trade-off. Without choice-trials, accuracy for high-reward (98.80% ± 0.19%) and low-reward targets (98.17% ± 0.33%) was at the same level. Responses to high-reward targets were not affected by choice-trial proportion (25: 99.24% ± 0.28%; 75: 98.74% ± 0.43%), whereas accuracy for low-reward targets decreased with increasing choice-trial proportion (25: 96.91% ± 0.56%; 75: 90.36% ± 1.52%).

In choice-trials, participants chose the high-reward target in 96.23% of trials, confirming that they indeed sought to maximize reward. Reaction times in choice-trials were faster in the 75% than in the 25% choice-trial condition (307 ms ± 7 ms vs. 328 ms ± 8 ms; t_(25)_ = 5.29, p < 0.001).

### Lateralized alpha and beta power

Lateralizations of alpha (8–14 Hz) and beta power (15–30 Hz) in the preparatory period of single-trials were analysed for a posterior and a centrolateral region of interest (ROI). Overall, alpha power decreased in both hemispheres in all choice-trial conditions. This was particularly pronounced in the parieto-occipital ROI (see Supplementary Fig. S1 for the parieto-occipital ROI and Supplementary Fig. S2 for the centrolateral ROI). To isolate more subtle differences between the hemispheres processing the low- or high-reward hemifield, lateralized power was calculated as the difference between contralateral and ipsilateral power with respect to the low-reward hemifield, scaled by the sum of activation of both hemispheres. The resulting index of lateralization varies from −1 to +1: positive values indicate greater power contralateral to the low-reward hemifield, negative values indicate the opposite pattern, and a value of 0 indicates the absence of hemispheric differences. Lateralized power estimates were baseline-corrected against the 500 ms preceding the preparatory period and averaged across the alpha and beta range for an early (100–300 ms) and a late (300–500 ms) time window of analysis in the 600 ms preparatory period. The first and last 100 ms of the preparatory period were excluded from analysis to ensure that it was not affected by perceptual processing of the fixation cross change (onset of the preparatory period) and of the target. The remaining 400 ms were split into an early and a late time window of analysis so that transient changes in lateralized power would not be missed.

**Figure 2:**
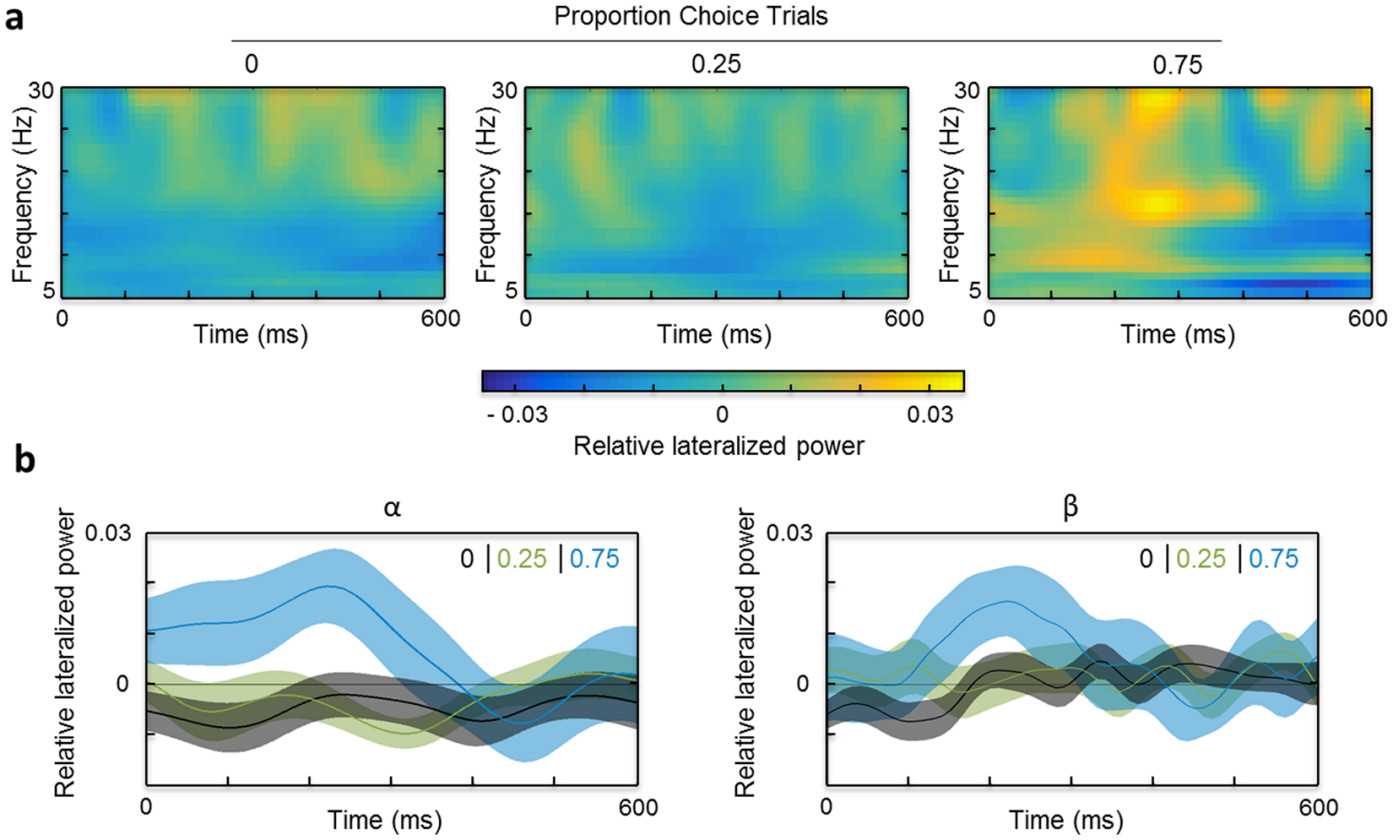
Lateralized power during the preparatory period: posterior ROI. (**a**) Time-frequency representations of the preparatory period in single-trials for the different proportions of choice-trials: 0 (left), 0.25 (middle) and 0.75 (right). Shown is relative lateralized power (baseline-corrected power contralateral minus ipsilateral with respect to the low-reward hemifield, scaled by the sum of power in both hemispheres) for frequencies of 5 to 30 Hz, for the 600 ms preparatory period preceding target presentation. Positive values indicate greater power contralateral than ipsilateral to the low-reward hemifield, negative values the opposite pattern, and a value of 0 indicates the absence of any hemispheric differences. (**b**) Relative lateralized alpha-band (8–14 Hz; left) and beta-band (15–30 Hz; right) power across the preparatory period, plotted separately for each proportion of choice-trials (0 in black, 0.25 in green, 0.75 in blue). Shaded areas show the standard errors of the means.

The results for the posterior ROI are plotted in Fig. 2: Fig. 2a shows the time-frequency representations (TFRs) for each choice-trial proportion, and Fig. 2b the relative lateralized power for each choice-trial proportion across the preparatory period averaged across the alpha-band and the beta-band. For posterior alpha, a main effect of time window (F_(1,25)_ = 4.57, p = 0.043, partial ƞ^2^ = 0.154) was observed, attributable to greater alpha power in the early time window of analysis (100–300 ms; 0.002 ± 0.003) than in the late time window (300–500 ms; −0.003 ± 0.003). Overall, there was no effect of choice-trial proportion, but a significant interaction with time window (F_(2,50)_ = 5.09, p = 0.01, partial ƞ^2^ = 0.169). While there was virtually no difference in alpha power between 0% and 25% choice-trial conditions in either time window (all M = −0.005, SE = 0.004, except for 25%, late time window: M = −0.005, SE = 0.003), lateralized alpha power increased in the early time window for the 75% condition (M = 0.016, SE = 0.007) as compared to the late time window (M = −0.001, SE = 0.007) and the other choice-trial conditions. For posterior beta, there was no effect of choice-trial proportion or time window, but the interaction fell just short of statistical significance (F_(2,50)_ = 3.10, p = 0.054, partial ƞ^2^ = 0.11). Similar to posterior alpha, lateralized posterior beta power increased in the early time window of the 75% choice-trial condition (M = 0.012, SE = 0.006; late time window and both time windows in the other choice-trial conditions: M = −0.001 to 0.001, SE 0 = 0.003 to 0.006).

**Figure 3:**
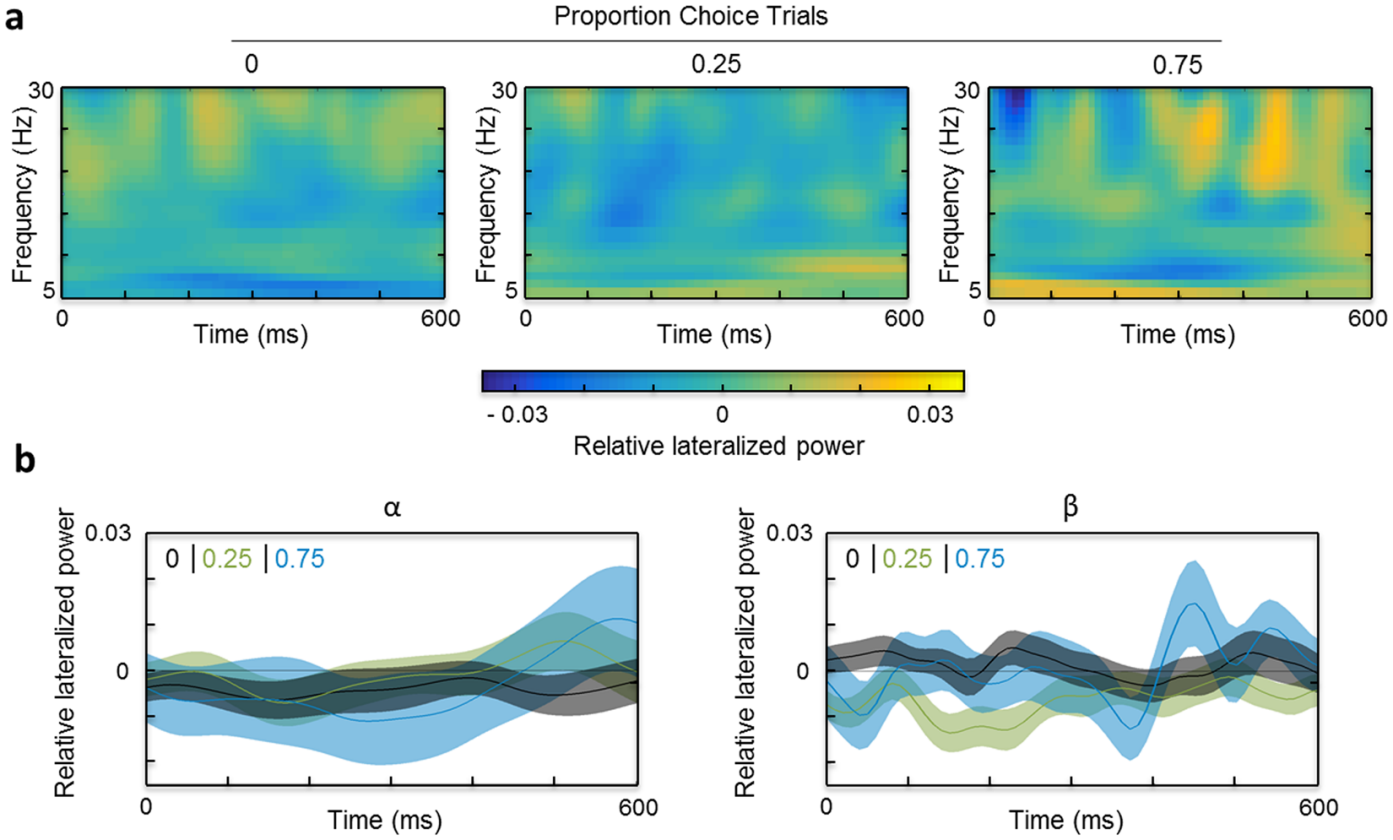
Lateralized power during the preparatory period: centrolateral ROI. (**a**) Time-frequency representations of the preparatory period in single-trials for the different proportions of choice-trials: 0 (left), 0.25 (middle) and 0.75 (right). Shown is relative lateralized power (baseline-corrected power contralateral minus ipsilateral with respect to the low-reward hemifield, scaled by the sum of power in both hemispheres) for frequencies of 5 to 30 Hz, for the 600 ms preparatory period preceding target presentation. Positive values indicate greater power contralateral than ipsilateral to the low-reward hemifield, negative values the opposite pattern, and a value of 0 indicates the absence of any hemispheric differences. (**b**) Relative lateralized alpha-band (8–14 Hz; left) and beta-band (15–30 Hz; right) power across the preparatory period, plotted separately for each proportion of choice-trials (0 in black, 0.25 in green, 0.75 in blue). Shaded areas show the standard errors of the means.

The results for the centrolateral ROI are plotted in Fig. 3, analogous to Fig. 2. There were no systematic changes in lateralized central alpha or beta power during the preparatory period, and no statistical effects reached significance. To ensure that no motor-level bias was missed, we also ran the same analysis for higher frequencies (30–50 Hz, i.e., the gamma band), and we checked for a Lateralized Readiness Potential (LRP) during the preparatory period (at electrodes C3 and C4). These analyses confirmed that there was no bias towards either effector.

### Correlations between lateralized power and behavioural measures

**Figure 4:**
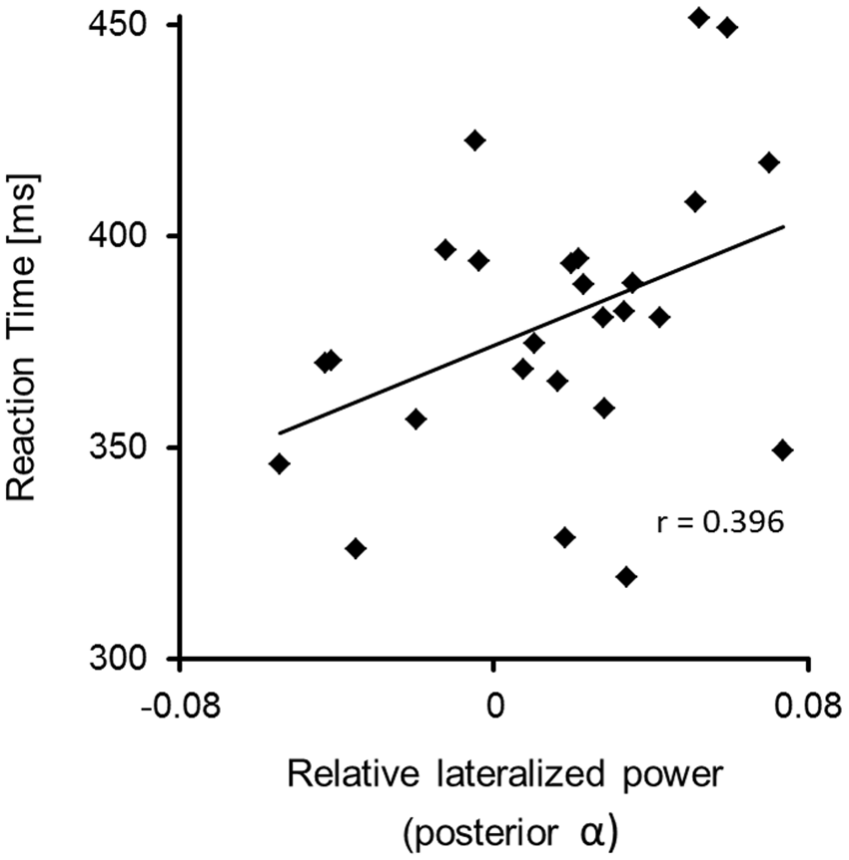
Correlation between lateralized posterior alpha power and reaction times. Shown are reaction times to low-reward targets in single-trials with 75% choice-trials in the same block as a function of relative lateralized posterior alpha power (baseline-corrected power contralateral minus ipsilateral with respect to the low-reward hemifield, scaled by the sum of power in both hemispheres) in the preparatory period.

Correlations were computed to test whether the observed effects in posterior alpha and beta power were behaviourally relevant and related to reaction times. Reasoning that the increase in lateralized alpha power with respect to the low-reward hemifield reflected increased suppression of that hemifield, we expected slower reaction times with greater pre-stimulus alpha power. We computed a correlation (Pearson’s correlation coefficient, *r*, one-tailed) between relative lateralized alpha power in the early time window of the 75% choice-trial condition and reaction times to low-reward targets in the 75% choice-trial condition. The scatterplot is shown in Fig. 4. Indeed, a positive correlation (r = 0.396, p = 0.023) indicated that the greater lateralized alpha power with respect to the low-reward hemifield in the preparatory period, the slower the reaction times to targets presented in that hemifield. The same correlation was computed for the lateralized beta power increase in the early time window of the 75% choice-trial condition, which was borderline significant (see section *lateralized alpha and beta power*). As we had no specific assumptions about the direction of a potential relationship between beta power over parieto-occipital cortex and behavioural measures, a two-tailed test was used for this correlation. Lateralized beta power with respect to the low-reward hemifield in the preparatory period was not related to response times to targets in that hemifield (r = 0.08, p = 0.699).

### Lateralized event-related potentials

**Figure 5:**
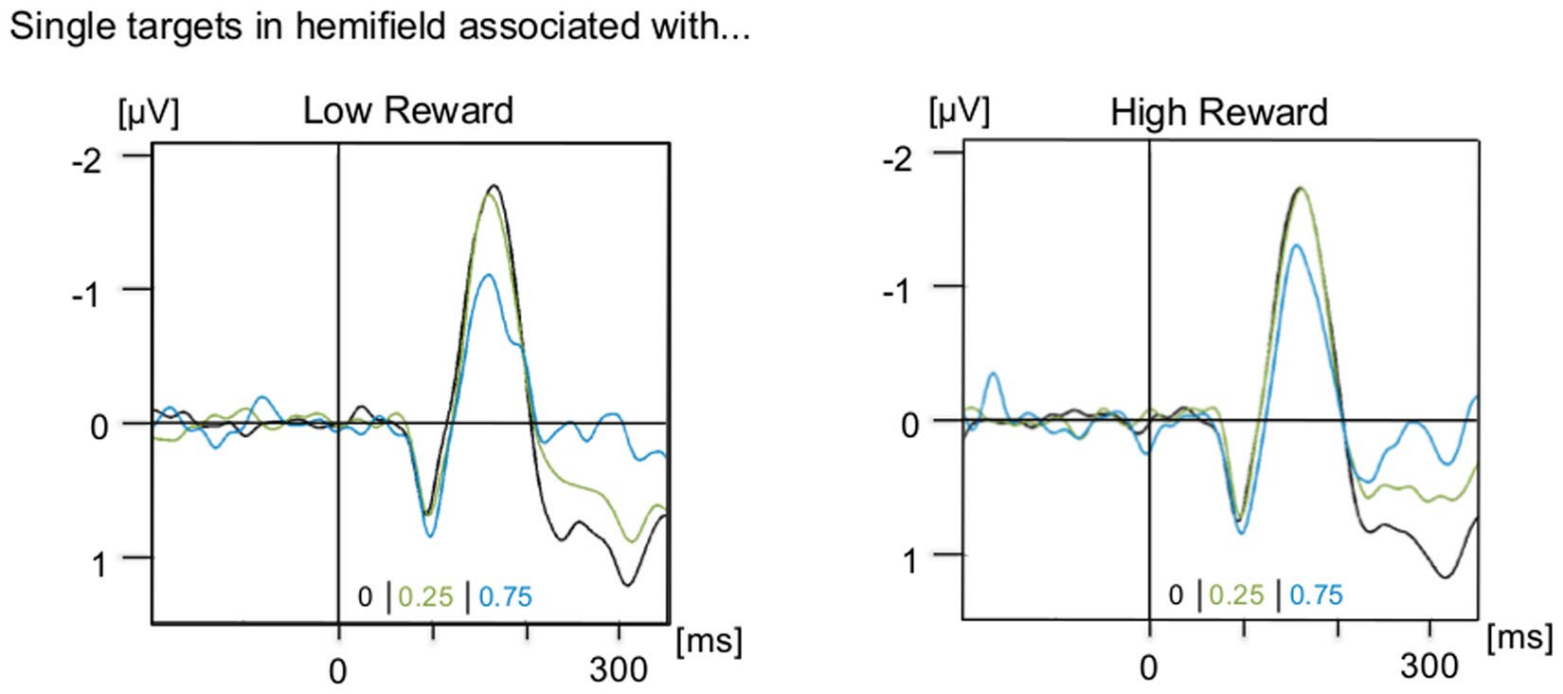
Grand-averaged lateralized ERPs (contralateral minus ipsilateral activity). The lateralized ERPs are shown time-locked to the onset of the target for parieto-occipital electrodes (PO3/4, PO7/8, O1/2), averaged separately for single targets presented in the hemifield associated with low-reward (left) and high reward (right), and for the different proportions of choice-trials in the same block (0 in black, 0.25 in green, 0.75 in blue). For illustration purposes, the waveforms were lowpass filtered at 35 Hz.

Choice-dependent reward effects on event-related potential components related to early perceptual processing (P1 and N1) were analysed by subtracting activity ipsilateral to the target from contralateral activity, separately for low- and high-reward targets and each choice-trial proportion. Mean amplitudes were computed for the lateralized P1 (70–120 ms) and N1 (120–220 ms). Lateralized event-related potentials time-locked to target onset are shown in Fig. 5. There were effects of choice-trial proportion for both lateralized P1 (F_(2,50)_ = 4.23, p = 0.02, partial ƞ^2^ = 0.145) and N1 (F_(2,50)_ = 16.89, p < 0.001, partial ƞ^2^ = 0.40, ɛ = 0.813). For the N1, pairwise comparisons revealed significant differences between the 75% and 0% choice-trial conditions (0.381 ± 0.08 μV, p < 0.001) and between the 75% and 25% choice-trial conditions (0.315 ± 0.08 μV, p = 0.002). Mean lateralized N1 amplitude was smaller in the 75% (−0.66 ± 0.16 μV) than in the 25% (−0.98 ± 0.19 μV) or 0% (−1.04 ± 0.19 μV) choice-trial conditions. More importantly, however, there were neither effects of reward magnitude nor interactions of choice-trial proportion and reward magnitude: the patterns of lateralized P1 and N1 amplitudes were equivalent for low- and high-reward single targets.

## Discussion

In this study, we investigated whether responses to single targets of different value are modulated by interleaved choices, and sought to identify a neural biasing mechanism underlying this modulation that takes effect during response preparation. The importance of the necessity to choose on reward effects has previously been demonstrated for saccadic eye movements: There was no effect of reward without interleaved choice-trials, and latencies of saccades to low-reward targets increased with increasing choice-trial proportion (Wolf *et al*., under review). In the present study, we observed a remarkably similar pattern of results for manual keypress responses, confirming that the effect of choice-trials on reward-related modulation of responses generalizes to other simple actions executed with different effectors. Only when choices had to be made within the same set of trials, reaction times were longer for low-reward targets than for high-reward targets. The magnitude of this response time delay increased with increasing choice-trial proportion. Taken together with the findings of Wolf *et al*. (under review), these results indicate that there might not be a direct influence of reward on response preparation, but that reward only affects behaviour when there is the necessity or possibility to optimize behaviour in order to maximize positive outcome. Here, expected value was only behaviourally relevant when choices could be made to maximize the received reward.

It may seem counterintuitive to view the reward-related bias in the present study as behavioural optimization, given that we observed behavioural costs. However, one should keep in mind that this cost was only observed in low-reward single-trials, in which only a small percentage of the available reward could be obtained (6.25% in the 25% choice-trial condition and 1.56% in the 75% choice-trial condition). Biasing processing in favour of the high-reward hemifield ensured optimal choices in choice-trials at only a small cost in single-trials, and thus served to maximize overall positive outcome.

Importantly, we do not claim that the opportunity to choose between options of different value is a necessary prerequisite for the observation of reward effects, but it is one way how reward can be assigned behavioural relevance. Another way behavioural relevance might be established is when selection of the target is more difficult than in the present study, such as in typical visual search tasks for which reward effects have repeatedly been observed without analogous choice opportunities^14,15,36,37^. Here, a target that is usually presented among (salient) distractors has to be localized and identified with temporal urgency. In this situation, when the risk of making a mistake and losing reward altogether is much higher than in the present task, biasing visual processing in a reward-related manner is often the most beneficial strategy to make sure that when mistakes are made, they are made with a low cost.

We analysed oscillatory brain activity in the preparatory period preceding target onset in order to identify a neural biasing mechanism underlying the effect of choices on reward-related response modulation. No bias in motor preparation, as indicated by lateralized oscillatory power in the beta-band over sensorimotor cortex (i.e., the centrolateral ROI) was observed, but the analysis revealed a relative posterior increase in alpha band power contralateral to the low-reward hemifield for a high choice-trial proportion (0.75). Lateralized increases in alpha power over parieto-occipital cortex have previously been reported to act as a spatially specific attentional suppression mechanism in preparation for locations that are to be ignored or selected against^21,25–28^. The present findings thus indicate that the low-reward hemifield was actively suppressed. Indeed, a correlation between relative lateralized alpha power with reaction times to the low-reward target in the 75% choice-trial condition confirmed that the greater the relative increase in alpha, the slower the responses to targets in the low-reward hemifield. These findings suggest that when oftentimes choices had to be made through which participants could effectively maximize positive outcome by choosing the target associated with a high reward, a bias in anticipatory selective attention served to suppress the low-reward hemifield in preparation for optimal target selection. This bias established prior to target onset resulted in delayed reaction times in low-reward single-trials, in which response to a target presented in the suppressed hemifield was required. Wolf *et al*. (under review) observed strong inter-trial effects: saccade latencies to the low-reward target in single-trials were delayed when following a choice for a high-reward target, but not when following a response to a single target in the high-reward hemifield. This might be taken to suggest that the overall delayed responses to low-reward targets with higher choice-trial proportions were due to lingering inhibition after a choice trial. But here, we observed a transient alpha power increase prior to target presentation that was baselined to the inter-trial period, but related to the responses to low-reward targets. These findings indicate that the delayed responses to low-reward targets were not the result of persistent inhibition from the previous trial, but that suppression of the low-reward hemifield only emerged during a preparatory period. This does not preclude that such a preparation is initiated after a choice- rather than after a single-trial, which would account for the n-1 effects reported by Wolf *et al*. (under review). With respect to the involvement of alpha oscillations in cognitive processes, our results add further support for conceptualizations of alpha emphasizing its inhibitory role^29,38,39^ and studies establishing an inverse relationship between posterior alpha power and behavioural measures of target perception in the contralateral hemifield^40–45^.

It should be noted that our finding of a *relative* contralateral alpha power increase in the 75% choice-trial condition does not imply that only the contralateral hemisphere was selectively modulated. The same finding could also be the result of an ipsilateral decrease or of both a contralateral increase and an ipsilateral decrease. It is tempting to compare contra- and ipsilateral activity in the 75% against the 0% choice-trial condition to clarify this issue, but this could be misleading. For instance, it is conceivable that there was an overall decrease in alpha power with increasing choice-trial proportion, reflecting a larger degree of preparation, which, combined with a low-reward-related contralateral increase in alpha power, could result in a pattern that would erroneously be interpreted as ipsilateral decrease. Simply put, there is no appropriate baseline to conclusively determine which hemisphere was modulated. But in any case, the lateralization indicates that there was a bias, and in light of the behavioural effects, we analysed the data with respect to the low-reward hemifield and interpret the finding as contralateral increase, reflecting suppression of this hemifield.

Notably, no lateralized posterior alpha power increase was observed in the 25% choice-trial condition, even though slower reaction times to low-reward targets as compared to the 0% choice-trial condition indicate that the low-reward hemifield was suppressed as well, arguably to a lesser degree than in the 75% choice-trial condition. The behavioural findings indicate a graded deployment of attention across the two visual hemifields consistent with the likelihood of getting an opportunity to decide against the low-reward target. Mixed results have been obtained as to whether a behaviourally evident graded deployment of attention reflects in posterior alpha activity. Gould *et al*.^32^ varied the validity of spatially predictive cues, and observed stronger lateralization of parieto-occipital alpha power with absolute (100% valid) than with slightly reduced spatial certainty (80% valid). No lateralization was observed for 60% cues, though. Of particular interest in the present context is a study by Dombrowe and Hilgetag^19^, who asked participants to either entirely shift attention to one hemifield, to balance it across the visual field, or to allocate more attention to one hemifield than the other (75% vs. 25%). Whereas posterior alpha power lateralized when attention was shifted to one hemifield, graded allocation of attention did not reflect in a lateralization. The graded condition of Dombrowe and Hilgetag^19^ seems to reflect the conditions with 75% and 25% choice-trials, and one might accordingly wonder why we observed a lateralization in one but not the other. However, when considering the entire trial context including single-trials, the likelihood of having to respond to a low-reward target was much higher in the 25% than in the 75% condition. In light of the previous findings, it is thus conceivable that in the 75% choice-trial condition, participants suppressed the low-reward hemifield, reflecting in lateralized posterior alpha and strongly delayed response times, and used a graded strategy in the 25% choice-trial condition, showing in delayed responses but not in lateralized alpha power changes. This line of reasoning might also explain why the lateralized alpha power increase was only transient and did not persist until target onset. Possibly, participants shifted from initial suppression of the low-reward hemifield (i.e., the early and transient alpha increase), to a slightly more graded deployment of attention over the course of the preparatory period. Relative distribution of attention might be reflected in top-down signals from frontal areas such as the frontal eye fields (FEF) to parieto-occipital cortex^19^, which have been specifically implicated in the control of spatial attention and modulation of anticipatory alpha activity^46–50^. This fits nicely with the fact that the FEF are also critical for overt visual selection, that is, they are involved in the selection and control of saccades and the representation of value in saccade tasks^51–53^.

In conclusion, we have provided further support for the notion that reward only affects response preparation to single targets when it is behaviourally relevant because of a necessity to optimize behaviour in order to maximize positive outcome. A mediation of reward effects on responses to single targets by interleaved choices between options associated with different levels of reward has previously been demonstrated for saccade preparation (Wolf *et al*., under review). Here, we show that this is a more general, effector-independent phenomenon. Preparatory lateralization of oscillatory alpha power over parieto-occipital cortex prior to target onset indicates that the delay in responses to low-reward single targets with a high proportion of choice-trials is the result of an anticipatory suppression of the low-reward hemifield. This is further supported by a correlation between the direction and magnitude of individual alpha lateralization and reaction times to low-reward targets.

## Methods

### Participants

Thirty-four students of Marburg University participated in the experiment. The experiment was approved by the Ethics Committee of the Faculty of Psychology at Philipps-University Marburg, and conducted in accordance with the ethical standards laid down in the Declaration of Helsinki. The data from eight participants had to be removed: four due to excessive oculomotor artefacts that contaminated >30% of all trials, and four due to technical issues during recording. The analyses were performed on the remaining twenty-six participants (twelve female, 14 male; mean age 23 years, range 20–29 years). All of them provided informed written consent prior to participation, were naive to the purpose of the experiment, and had normal or corrected-to-normal visual acuity and colour vision. Visual acuity and colour vision were tested with the OCULUS Binoptometer 3 (OCULUS Optikgeräte GmbH, Wetzlar, Germany).

### Apparatus and stimuli

Participants were seated in a comfortable chair in a dimly-lit and electrically shielded room. The monitor (22”, 1680 × 1050 px) was placed at a viewing distance of 104 cm. Stimulus presentation and response collection were controlled by a Windows PC using E-Prime 2.0 software (Psychology Software Tools, Inc.). Participants responded by pressing buttons on the back of a gamepad (Microsoft SideWinder USB).

All stimuli were black and presented against a grey background. The circle-shaped targets subtended 0.55° of visual angle and appeared at an eccentricity of 9.84° from fixation. The placeholders and the small fixation cross shown during the preparatory period and during target presentation were of the same size as the target (0.55°). The large fixation cross presented at the beginning of each trial and in-between trials, and the reward feedback presented at the end of each trial subtended 1.10°.

### Procedure and Design

The trial procedure is illustrated in Fig. 1a. Each trial started with the presentation of the fixation cross for a duration between 500 and 1000 ms (in steps of 100 ms, randomly chosen in each trial). Two crosses (placeholders) were present to the left and right of fixation throughout the experimental trials. The central fixation cross then changed its size to indicate the onset of the target after another 600 ms (*preparatory period*). In single-trials, one single target appeared at one of the locations of the placeholders with equal frequency, and participants were to indicate whether the target was presented left or right from fixation by pressing the left (left target) or right (right target) button on a gamepad with their left or right index finger. In choice-trials, a target appeared at both placeholder locations, and participants were free to choose to respond to either one with a left or right button press. The target display was present until response, but for a maximum of 700 ms. Rewards were given after correct responses within the reaction time window of 700 ms. In each block of trials, one hemifield was associated with a high and the other with a low reward. In single-trials, correct responses within the reaction time window were rewarded with either a high (+9 points) or a low reward (+1 point), depending on the hemifield of target presentation. If participants responded incorrectly or not within the reaction time window, they received no reward (+0 points). In choice-trials, participants received the reward associated with the hemifield of the chosen target. Reward points were converted into monetary reward at the end of the experiment (1 € for 2000 points). The reward feedback display was presented for 700 ms. The interval between trials varied randomly between 500 and 1000 ms, in steps of 100 ms.

The experiment consisted of 2304 trials in total. The three proportions of choice-trials (0 vs. 0.25 vs. 0.75) were crossed with the assignment of reward to the two hemifields (left high, right low vs. left low, right high) and varied blockwise, yielding six blocks of 384 trials each. Participants were informed about this design. The order of blocks was balanced across participants. Every 48 trials, participants were given the opportunity of a short rest.

### Behavioural analyses

Trials with excessively long reaction times (>2.5 SD from mean reaction time, calculated separately for each participant; on average, 2.6% of all trials) were removed from the data. Mean reaction times in single-trials were analysed for each participant, separately for each proportion of choice-trials (0 vs. 0.25 vs. 0.75) and for high- and low-reward targets and submitted to a two-way repeated measures ANOVA. Only correct responses were included. For these and all other analyses, normality of the data was checked by visually inspecting Q-Q-plots. Sphericity was tested (Mauchly’s test) but never violated. Accuracy in single-trials was checked to ensure that speed-accuracy trade-offs did not contribute to any differences in reaction times. Additionally, the proportion of high-reward choices in choice-trials was computed as a manipulation check.

### EEG recording and analyses

The EEG was recorded from 64 Ag/AgCl electrodes (actiCAP, Brain Products, Munich, Germany) positioned according to the international modified 10–20 system. Vertical (vEOG) and horizontal electrooculogram (hEOG) were recorded as the voltage difference between electrodes positioned above and below, and to the left and right of the eyes, respectively. Electrode impedances were kept below 5 kΩ. The signal was recorded with a BrainAmp amplifier (Brain Products) at a sampling rate of 1000 Hz with a high cutoff filter of 250 Hz and a low cutoff filter of 0.016 Hz. All electrodes were referenced to FCz and rereferenced offline to the average of all electrodes.

EEG preprocessing and analyses were performed in Matlab (MathWorks) using the Fieldtrip toolbox^54^ and custom scripts. For analysis of oscillatory activity in the preparatory period of single-trials as a function of the proportion of choice-trials, the continuous EEG was segmented into epochs of 2200 ms, starting 1000 ms prior to the onset of the preparatory period and ending 1200 ms thereafter. This long epoch was chosen to allow for the calculation of wavelet coefficients for all frequencies and time points of interest (i.e., the preparatory period and a baseline period from −500 to 0 ms). For analysis of post-stimulus event-related potentials to the target, the EEG was segmented into epochs of 550 ms, starting 200 ms prior to target onset. The time period from −200 ms to 0 was used for baseline correction. Trials with response errors, trials identified as reaction time outliers (>2.5 from mean RT calculated separately for each participant) and trials with blinks (vEOG > 100 μV) or eye movements (hEOG > 70 μV) in the critical time windows (−500 to 600 ms with respect to the onset of the preparatory period and −200 to 350 ms with respect to target onset) were excluded. Additionally, segments were excluded from further analysis when the absolute voltage in the channels of interest (FC3/4, C3/4, CP3/4, PO3/4, PO7/8 and O1/2 for the preparatory period; PO3/4, PO7/8 and O1/2 for target processing) exceeded 80 μV.

TFRs of the preparatory period in each trial were computed by convolving 5-cycle Morlet wavelets with the EEG data segments for frequencies from 5 to 30 Hz, with a resolution of 1 Hz. This procedure was applied in steps of 10 ms from −500 ms to 600 ms relative to the onset of the preparatory period for six electrode-pairs in ROIs: one posterior (PO3/4, PO7/8, O1/2) and one centrolateral (FC3/4, C3/4, CP3/4). The TFRs were sorted into trials in which low reward was associated with the left hemifield and trials in which low reward was associated with the right hemifield. Channels were defined as contralateral or ipsilateral with respect to the low-reward hemifield and averaged separately for the two ROIs. Lateralized power was then calculated as the difference between contralateral and ipsilateral wavelet coefficients ω at time point t, scaled by the sum of activation of both hemispheres, averaged across trials in which low reward was associated with the left and right hemifield.

1 $$\begin{array}{c}{\rm{Lateralized}}\,{\rm{power}}=(({\rm{low}}\,{\rm{reward}}\,{\rm{left}}\frac{{{\rm{\omega }}}_{{\rm{t}}}({\rm{contralateral}})-{{\rm{\omega }}}_{{\rm{t}}}({\rm{ipsilateral}})}{{{\rm{\omega }}}_{{\rm{t}}}({\rm{contralateral}})+{{\rm{\omega }}}_{{\rm{t}}}({\rm{ipsilateral}})})\\ \quad \quad \quad \quad \quad \quad \quad \quad \,+({\rm{low}}\,{\rm{reward}}\,{\rm{right}}\frac{{{\rm{\omega }}}_{{\rm{t}}}({\rm{contralateral}})-{{\rm{\omega }}}_{{\rm{t}}}({\rm{ipsilateral}})}{{{\rm{\omega }}}_{{\rm{t}}}({\rm{contralateral}})+{{\rm{\omega }}}_{{\rm{t}}}({\rm{ipsilateral}})}))/2\end{array}$$

This procedure yields an index of lateralization that varies from −1 to +1, with positive values indicating greater power contralateral to the low-reward hemifield, negative values indicating the opposite pattern, and a value of 0 indicating the absence of any hemispheric differences in power. Equivalent or similar indices of lateralized power have been used before to track the deployment of spatial attention and motor preparation^24,31,43,55,56^. Lateralized power estimates were baseline-corrected by subtracting the average lateralized power in the 500 ms preceding the onset of the preparatory period at each frequency. The resulting relative lateralized power estimates were averaged across frequency bins in the alpha (8–14 Hz) and in the beta range (15–30 Hz) for an early (100–300 ms) and a late (300–500 ms) time window of analysis in the preparatory period, separately for the two ROIs. Alpha and beta power estimates in each ROI were then submitted to a two-way repeated measures ANOVA with the factors proportion of choice-trials (0 vs. 0.25 vs. 0.75) and time window of analysis (early vs. late).

For post-stimulus analysis of perceptual processing of the target, event-related potentials were calculated for parieto-occipital electrodes (PO3/4, PO7/8, O1/2) time-locked to the onset of the target. Channels that were contralateral or ipsilateral to the target were averaged separately for trials in which the target was presented in the hemifield associated with low reward and for trials in which the target was presented in the hemifield associated with high reward. Difference waves were calculated by subtracting ipsilateral from contralateral activity. Mean amplitudes were computed for the time windows from 70 to 120 ms (P1) and from 120 to 210 ms (N1). These time windows of analysis were determined based on visual inspection of the grand average across all participants and conditions. Two-way repeated measures ANOVAs with the factors proportion of choice-trials (0 vs. 0.25 vs. 0.75) and reward magnitude (low vs. high) were computed separately for P1 and N1 time windows.

## Electronic supplementary material

Supplementary Information
