# Supplementary material for: The necessity to choose causes reward-related anticipatory biasing: Parieto-occipital alpha-band oscillations reveal suppression of low-value targets
Source: Sci Rep. 2017 Oct 30;7:14318. doi: 10.1038/s41598-017-14742-w (PMC5662762; doi:10.1038/s41598-017-14742-w)
Supplement: Supplementary file 1 — Supplementary Information [file 41598_2017_14742_MOESM1_ESM.pdf]

The necessity to choose causes reward-related anticipatory biasing:

Parieto-occipital alpha-band oscillations reveal suppression of low-value targets

Anna Heuer[1*], Christian Wolf[1], Alexander C. Schütz[1], & Anna Schubö[1]

[1]Experimental and Biological Psychology, Philipps-University Marburg, Marburg Germany

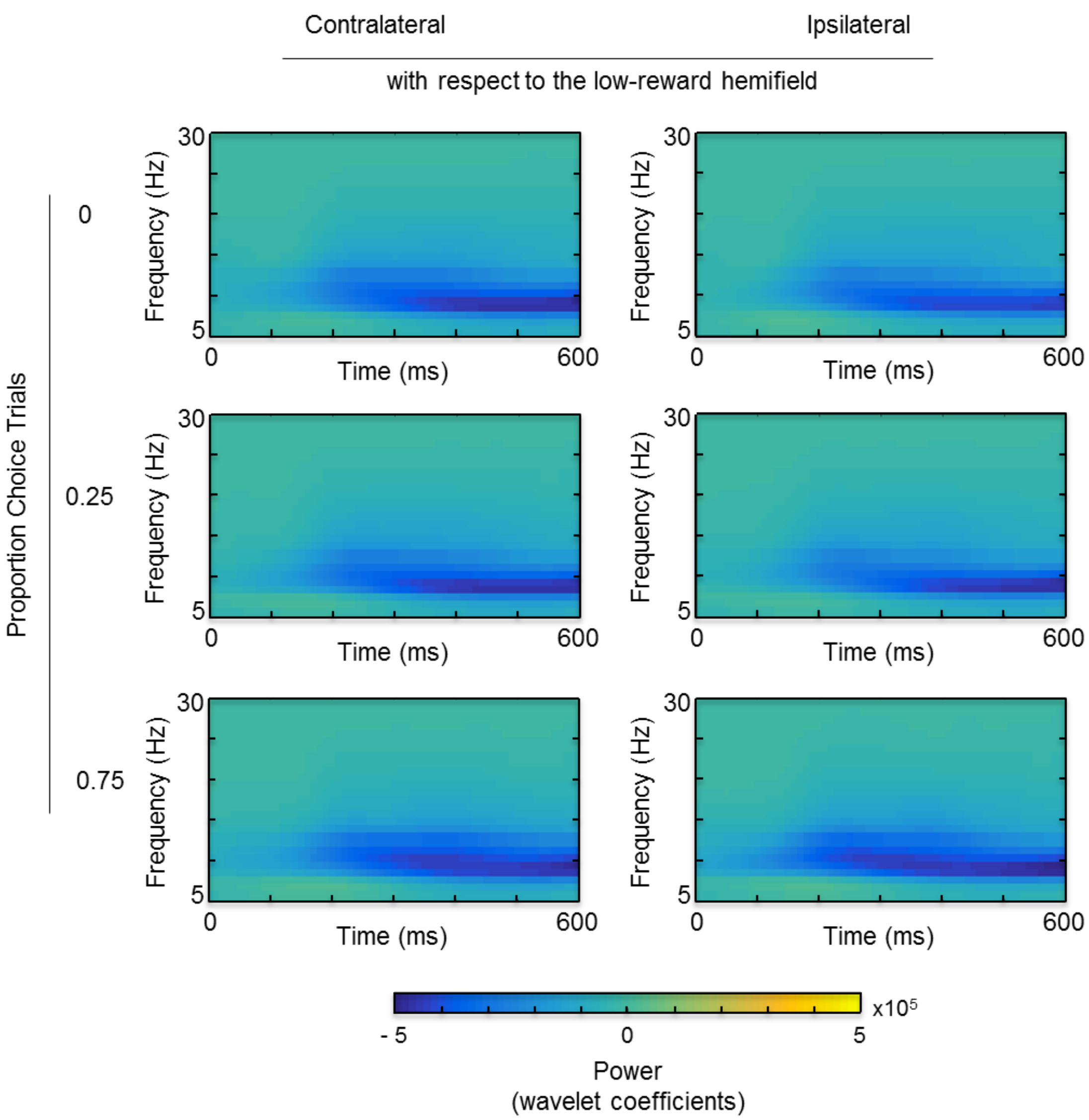

Supplementary Figure S1. Contra- and ipsilateral power during the preparatory period: posterior ROI. Time-frequency representations of the preparatory period in single-trials for the different proportions of choice-trials (0 in the top row; 0.25 in the middle row and 0.75 in the bottom row), shown separately for the contralateral (left column) and ipsilateral (right column) hemisphere with respect to the low-reward hemifield. Shown is the power (i.e., the wavelet coefficients) for frequencies of 5 to 30 Hz, for the 600 ms preparatory period preceding target presentation.

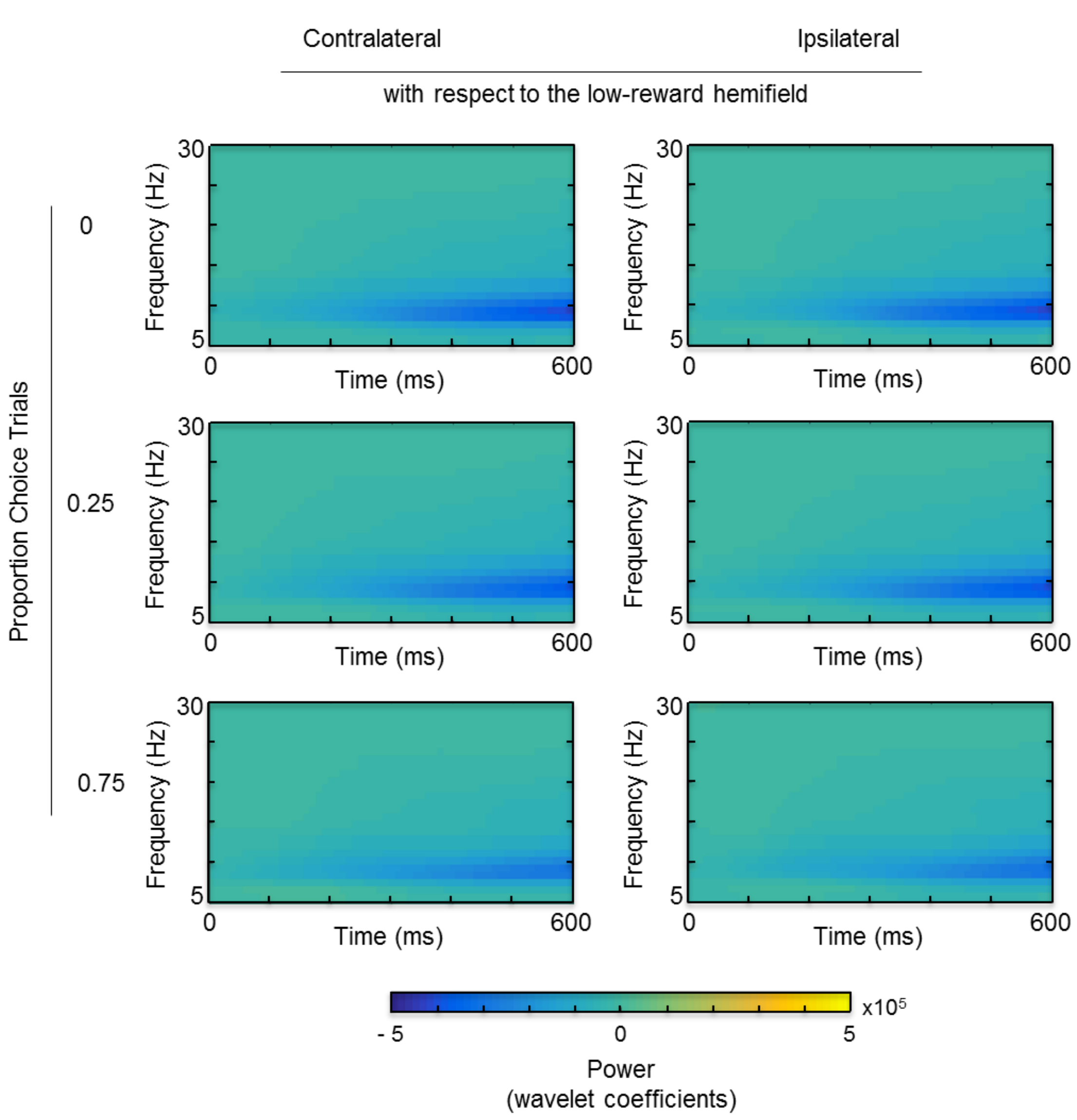

Supplementary Figure S2. Contra- and ipsilateral power during the preparatory period: centrolateral ROI. Time-frequency representations of the preparatory period in single-trials for the different proportions of choice-trials (0 in the top row; 0.25 in the middle row and 0.75 in the bottom row), shown separately for the contralateral (left column) and ipsilateral (right column) hemisphere with respect to the low-reward hemifield. Shown is the power (i.e., the wavelet coefficients) for frequencies of 5 to 30 Hz, for the 600 ms preparatory period preceding target presentation.
